# Surface Roughening Behavior of the 6063-T4 Aluminum Alloy during Quasi-in Situ Uniaxial Stretching

**DOI:** 10.3390/ma15186265

**Published:** 2022-09-09

**Authors:** Yang Cai, Xiaosong Wang, Yan Du

**Affiliations:** 1 School of Materials Science and Engineering, Harbin Institute of Technology, Harbin 150090, China; 2 School of Materials Science and Engineering, Heilongjiang University of Science and Technology, Harbin 150022, China

**Keywords:** surface roughening, orange peel, uniaxial stretching, in situ-EBSD, polycrystal aluminum alloy, deformation coordination

## Abstract

Owing to orange-peel defects, the industrial application of light alloy structural members is significantly restricted. In this study, a quasi-in situ axial tensile experiment was conducted on a 6063-T4 aluminum alloy sample. The surface morphology and microstructure evolution of the tagged area were scanned simultaneously using laser scanning confocal microscopy and electron backscattered diffraction, and the surface roughening behavior of the polycrystal aluminum alloy surface, caused by deformation, was quantitatively analyzed. As the concave–convex features at the surface appear in pairs with increasing global strain, the width of the concave features increases, whereas that of the convex features decreases gradually, resulting in the initially increasing surface roughness, which subsequently remains unchanged. During the stretching process, the small-sized grains in the 37~102 μm range show weak strain localization and the highest coordination of deformation. The deformation mode of medium-sized grains in the 114–270 μm range tends to grain deflection, and others tend to slip.

## 1. Introduction

**Figure 1:**
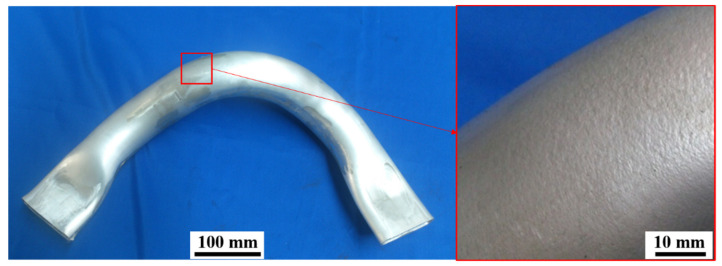
Aluminum alloy structural member and “orange-peel” defects.

The production of lightweight components has become a point of discussion in advanced manufacturing. They have been developed for the application in new energy vehicles that help reduce energy consumption and realize resource economization and efficiency [1,2]. Orange-peel defects, which are surface defects, are often observed on the surfaces of lightweight components, such as aluminum, magnesium, and titanium [3,4], as shown in Figure 1. Distinct deteriorations in surface roughness [5], surface performance [6], weldability, fatigue life [7,8,9], and even forming limits [10,11] arise from roughened surfaces. Thus, the application of light alloy components in the industry is considerably restricted.

Surface roughening is a commonly occurring surface phenomenon with a series of concave–convex features and is caused by plastic deformation. It is known as a surface orange-peel defect when the surface roughening sufficiently grows to a macroscopically visible defect [12]. The surface roughening of polycrystalline alloys [13] is affected by two aspects: plastic deformation (including stress and strain state [14], stress–strain path [15], strain rate, and work-hardening ability) and microstructure (grain size, grain orientation, texture, microdefects [16], and other factors).

Experiments have demonstrated that the surface roughness increases with increasing plastic strain [17], or first increases then gradually decreases at a maximum roughness [18] for pure copper and pure aluminum. The evolution trend of surface roughness is determined by the coordination deformation ability that predominantly depends on grain size. Generally, the strong matrix texture and grain refinement are both advantageous for deformation coordination [19,20].

Fundamentally, the formation of each bump or pit on a rough surface is closely related to the slip system, precipitations, and inclusions of the grains. The peak appears near the grain boundary, where it is difficult for slippages occurring [21]. The presence of inclusions in a subsurface layer also affects the surface features, and bumps and pits form above the hard and soft inclusions, respectively [22,23]. The height of the surface undulations is affected by the inclusion-to-surface distance [24]. Soft-oriented grains are more prone to plastic deformation than hard-oriented grains [25].

In addition, the coordination deformation capability is also related to the strain state, such as uniaxial stretching, biaxial stretching [26], and bending deformation [27], and results in different strain localization. The degree of surface roughness produced in the biaxial stress state is significantly less than that in the uniaxial stress state [28,29,30]. The strain localization is a primary factor that induces surface roughening [31,32].

However, few studies have analyzed the quantitative relationship between macroscopic surface roughness and strain, dynamic evolution between surface roughening behavior, and grain-size effect. In this study, the surface morphology and microstructure of an aluminum alloy sample were observed via quasi-in situ tensile experiments. The surface morphology evolution process and corresponding microstructure were both quantificationally investigated. The dimensional variations of bumps and pits were qualitatively discussed, and the grain-orientation variations and strain localization were quantitatively analyzed in every size range in this study, thereby laying a theoretical foundation for avoiding orange-peel defects on the surfaces of light alloy components.

## 2. Experiments

### 2.1. Quasi-in Situ Tension Testing

**Figure 2:**
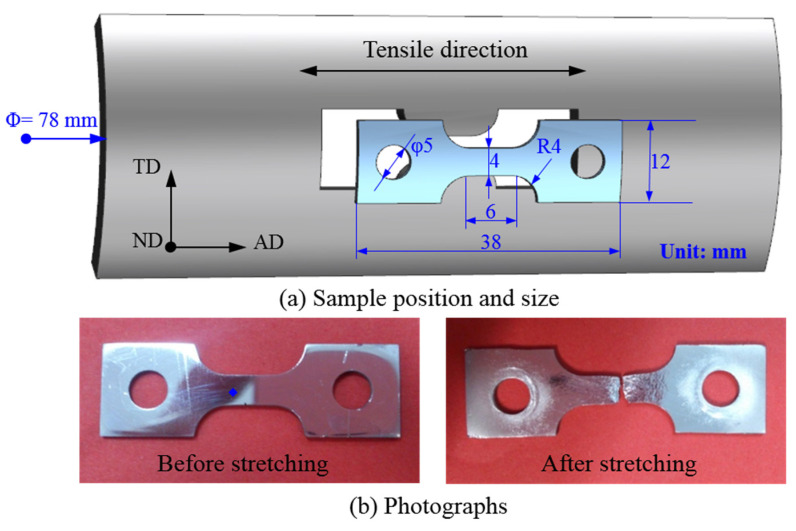
(**a**) Sample position and size. (**b**) Photographs of quasi-in situ sample.

To study the formation mechanism of surface roughening behavior and the phenomenon of concave–convex features caused by the plastic deformation of polycrystal aluminum alloy, a quasi-in situ tensile experiment was conducted. The experimental material was a 6063-T4 aluminum alloy extruded tube. The axial direction of the tube is denoted as AD, the tangential direction as TD, and the normal direction as ND. The in situ tensile specimens were prepared using electrical discharge machining wire cutting along the AD of the tube, and the thickness along the ND was 1 mm. The size and sampling direction are shown in Figure 2a, and the available tensile area of the specimen is 6 × 4 mm. The tensile test was performed using an electronic universal material testing machine (Instron 5569R, Norwood, MA, USA) at a stretching velocity of 1 mm/min at 25 °C. The strain rate negligibly influenced the microstructure at a temperature in the range of −20 °C to 20 °C [33]. The brief unloading was performed at tensile deformations of 2.14%, 6.32%, 9.62%, and 14.12% for SEM and EBSD testing. Photographs of the in situ tensile specimens before and after stretching are shown in Figure 2b, showing that the specimen surface resembles a mirror before stretching, and then becomes a rough surface after stretching.

### 2.2. Surface Roughness Characterization

The surface roughness (*R_a_*) was characterized within an area of 2560 × 2560 μm to reasonably represent surface roughening and was tested using laser scanning confocal microscopy (OLS 3000, Tokyo, Japan). The magnification of the microscope ranges from 120× to 14,400×, and it was used to characterize the surface morphology and surface roughness at different strain stages. The surface roughness, $R_{a}$, of the sample is the arithmetic average of the roughness profile [34]:

(1) $$R_{a}=\frac{1}{l}\int_{0}^{l}\left|f\left(x\right)\right|dx$$

where l is the sampling length and f(x) is the offset distance from the profile within the sampling length. The surface roughness of the polished surface was measured before stretching and was observed to be 0.25 μm. It is considered that the surface roughness of a mirror surface does not exceed 0.4 μm; therefore, it can be assumed that the surface of the sample in this experiment was a mirror before stretching [35].

### 2.3. Microstructure Characterization

Electron backscattered diffraction (EBSD) was performed using the field emission environmental scanning electron microscopy (FE-SEM, Quanta 200FEG, FEI, Hillsboro, OR, USA) with a step size of 5.5 μm. Before EBSD analysis, the tensile specimens were electropolished. The electrolyte ratio was 10% HClO_4_ to 90% C_2_H_5_OH, the temperature was approximately −20 °C, the voltage was 15 V, and the polishing time was approximately 50 s. However, the SEM and EBSD areas were not selected as the same area, and only the changes in surface morphology and microstructure were observed.

## 3. Results and Discussion

### 3.1. Surface Morphology

**Figure 3:**
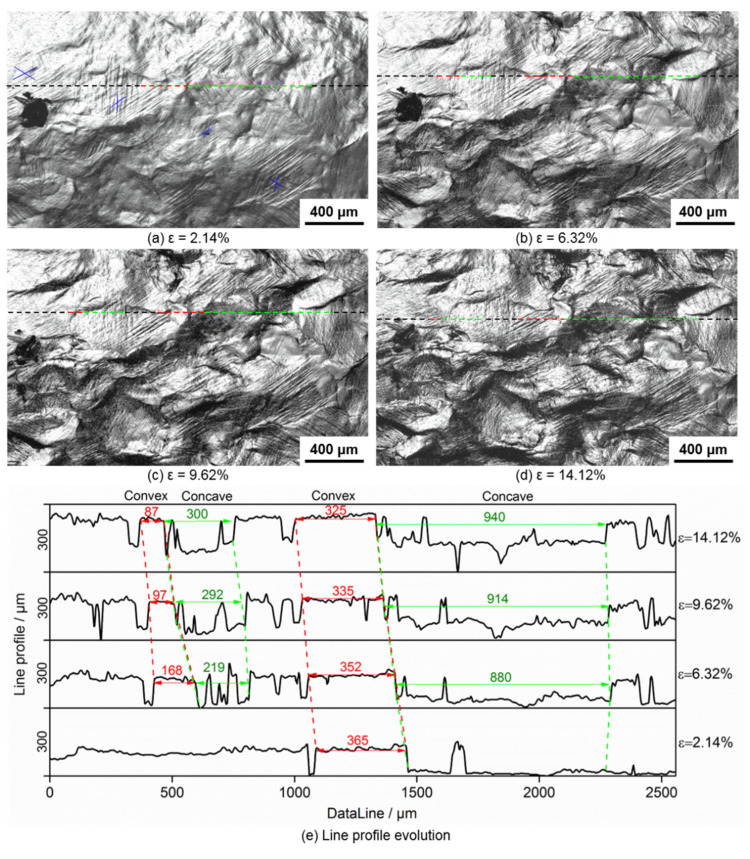
Surface morphologies of the marked area when the sample was stretched to global strains of (**a**) 2.14%; (**b**) 6.32%; (**c**) 9.62%; (**d**) 14.12%; and (**e**) line profile evolution.

A significant advantage of in situ-SEM is the ability to show the real-time corresponding relations between the concave–convex features, slip lines, and global strain. The line profiles can be obtained by 2D images using laser scanning confocal microscopy. Figure 3a–d shows that the concave and convex features simultaneously and continuously appear in the surface morphology. The corresponding positions are predominantly formed with shallow concave features at the initial deformation stage and are indicated using the green dashed line, and adjacent concaves are indicated using red lines. As stretching progresses, the concave and convex features appear more distinctly. Numerous slip traces (indicated by blue “X”) are observed at a global strain of 2.14%, as shown in Figure 3a. There are few or no slip traces in certain areas, but there are marked or even cross slips in other areas. This signifies that the distribution of plastic deformation is non-uniform in the initial stage of plastic deformation [36].

Figure 3e shows the line profile evolution of the sample at different strain stages. The line is scanned along the tensile direction and the length is 2560 μm. The line profile appears to have undergone profile changes, with five turning points that divide the concave and convex features, and the maximum height of the undulation is approximately 150 μm when the global strain is 2.14%. The new concave–convex features appear, and the maximum height of the undulation increases to approximately 170 μm when the global strain reaches 6.32%. Then, a few new concave–convex features appear when the deformation exceed 9.62%. Line scanning evolution shows that the widths of the concave and adjacent convex features increase and decrease, respectively. The maximum height of the undulation increases at strains of 6.32% and 9.62% and then remains virtually constant at a strain of 14.12%.

### 3.2. Surface Roughness

**Figure 4:**
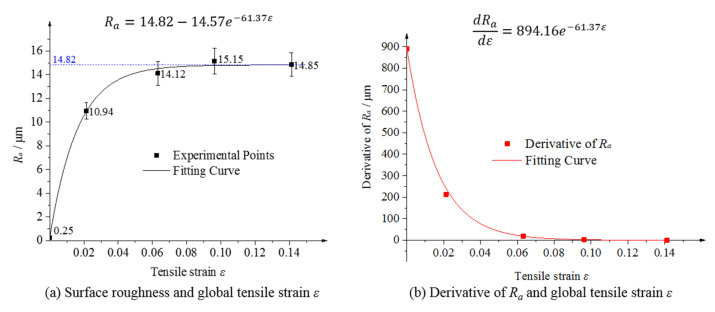
Surface roughness of the sample at different strain stages.

The resulting surface roughness is a macroscopic manifestation of the microscopic concave and convex features. The surface shifted from a mirror finish to a rough surface owing to deformation. To measure the surface roughness of the deformation-induced rough surface, the surface roughness, *R_a_*, of the line profile was calculated using Equation (1) at different tensile strains, as shown in the experimental points of Figure 4a. It is true that surface roughness variation must be continuous due to the continuity of deformation. From the view of experimental points, the surface roughness, *R_a_*, first increases and then remains unchanged with increasing tensile strain. It can be observed that the gradient of the *R_a_* shows an obvious transformation. To research the variation of *R_a_* deeply and quantificationally, the derivatives of *R_a_* were calculated and shown in Figure 4b. It is obvious that the derivatives of *R_a_* were exponentially dependent on the tensile strain. The fitting type is the exponential fit by Origin software, refer to the literature [28], and the R-squared value is 0.99 showing a higher confidence coefficient. It shows that the derivative drops rapidly from a high value of 900 to almost 0. That means the surface roughness increases rapidly at the beginning and then stays at the same level. Secondly, the mathematical expression for surface roughness, *R_a_*, is obtained by integrating the derivative, as shown in Figure 4a. The obtained integral curve fits well with the experimental points. There are deviations between the fitting curve and the experimental points, where the maximum deviation is 0.4 μm when the strain is 6.32%, and the other deviations are all within 0.3 μm. In conclusion, with increasing tensile deformation, the surface roughness, *R_a_*, first increased rapidly and then remained at the same level.

### 3.3. Microstructure

To study the microscopic evolution process of surface roughening on polycrystal aluminum alloy during stretching, an in situ-EBSD test was performed, and the dynamic microstructure evolution process at the sample surfaces was obtained. The microstructure evolution process was studied with respect to the aspects of grain-orientation variation and strain localization.

#### 3.3.1. Grain-Orientation Variation

**Figure 5:**
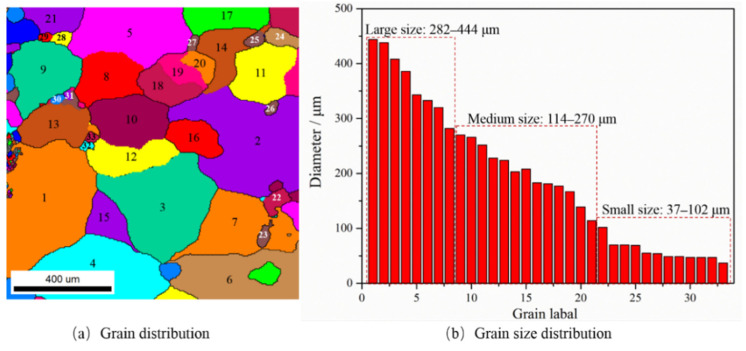
Grain and their grain-size distribution observed by EBSD.

There were 33 grains with grain sizes between 37 and 444 μm in the observable field, except for some grains, such as the grains with incomplete boundaries or those that are considerably small. We considered the grain sizes of 282–444 μm as large grains, 114–270 μm as medium grains, and 37–102 μm as small grains, and their grain distribution and grain-size distribution are shown in Figure 5. Surface roughening is initiated by the deflection of the surface grains relative to the original surface during plastic deformation. Therefore, to characterize the correlation between surface roughening behavior and grain size, the deflection degrees of the large, medium, and small grains were separately analyzed.

**Figure 6:**
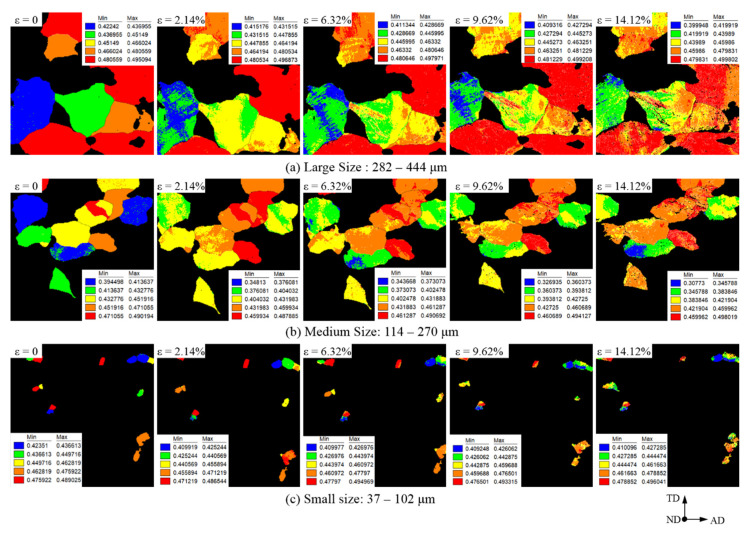
Schmidt factor distribution of the observable field observed by EBSD.

The grain orientations of the surface grains, with random Schmidt factors, were randomly distributed in the initial state and then inevitably deflected with stretching. The Schmidt factor was used to characterize the grain orientation, and the deflection of the surface grains was reflected via the change in the Schmidt factor. The Schmidt factor distribution of the observable field is shown in Figure 6. The Schmidt factors of these 33 grains were randomly distributed in the 0.43–0.50 range before stretching; after stretching, the Schimidt factors of the grains changed. The change in the Schmidt factor increased with increasing tensile deformation. Even the deformation of some small-sized grains was too severe, where it could not be recognized when the deformation was 14.12%.

**Figure 7:**
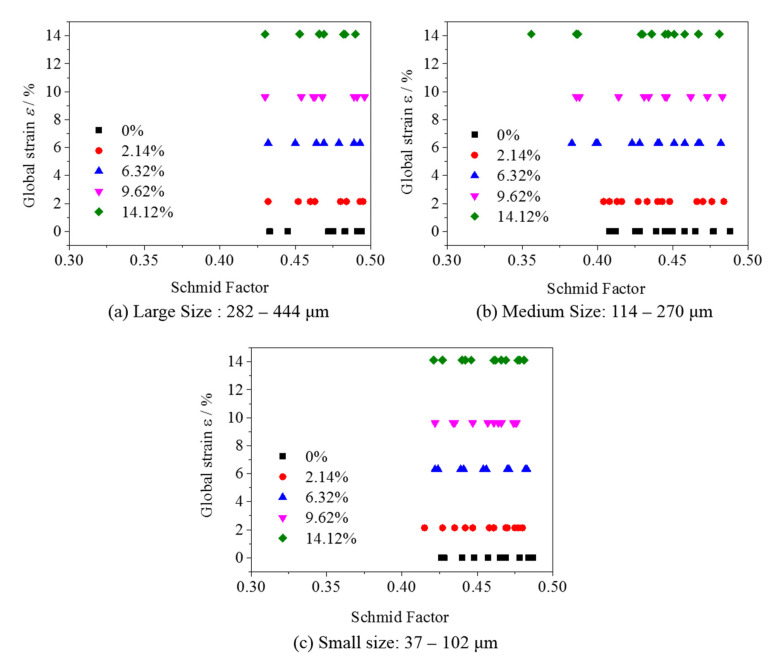
Statistics of Schmidt factors of the observable field observed by EBSD.

To quantitatively characterize the degree of deflection of surface grains of different sizes, the average value of the Schmidt factor for each grain was calculated and shown in Figure 7. The deflection degree of the large-sized grains is the smallest, that of the small-sized grains is medium, and that of the medium-sized grains is the largest. Medium-sized grains, i.e., a grain size of 114–270 μm, were prone to be deflected during stretching. The Schmidt factor changes toward smaller values until 0.36.

#### 3.3.2. Strain Localization

**Figure 8:**
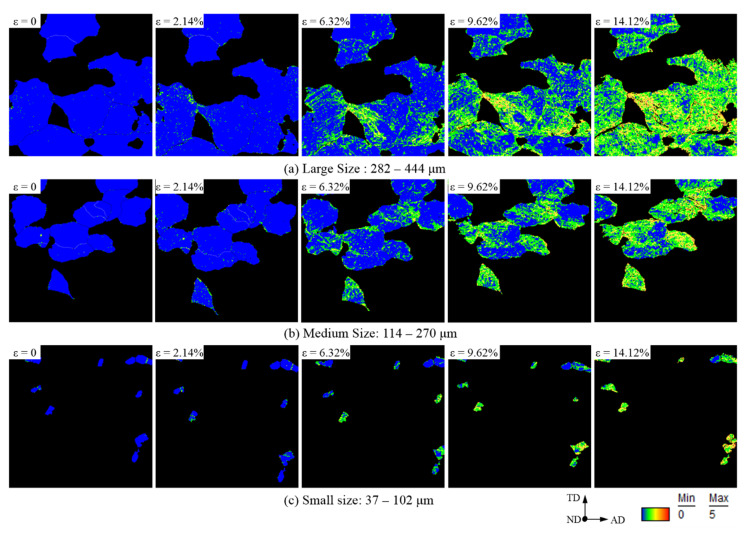
KAM distribution of the observable field observed by EBSD.

The kernel average misorientation (KAM) at different strains is shown in Figure 8; the strain localization degrees were indicated using different colors. Red represents the most severe plastic deformation, that is, the highest degree of strain localization. Orange represents the second-most severe, yellow represents the general degree, green represents less plastic deformation, and blue indicates no plastic deformation. Geometrically necessary dislocations (GNDs) are dislocations generated to maintain the continuity of crystal deformation and can be represented by KAM [37].

**Figure 9:**
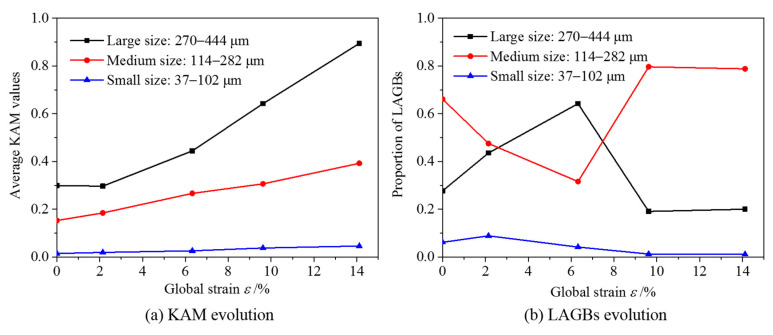
KAM and LAGBs evolutions of the observable field observed by EBSD.

As observed in the KAM distribution, a global strain of 2.14% already induces strain localization. The deformation predominantly occurs at the grain boundaries and then spreads from the grain boundary into the grain with increasing global strain. The quantitative statistics of the KAM are shown in Figure 9a and show a strong regularity. The geometrically necessary dislocations, which are expressed using KAM, increase with increasing strain. The KAM increases the fastest in large-sized grains, indicating that the GND in large-sized grains increases most distinctly, and the deformation coordination is moderately weak during the axial stretching process. Accordingly, the deformation coordination in the small-sized grains is the highest.

In general, numerous microcosmic defects in the polycrystalline microstructure can be caused by plastic deformation, and these defects can be represented by the generation of lower-angle grain boundaries (LAGBs, 2–15°). The quantitative statistics of the content of LAGBs are shown in Figure 9b, which shows its correlation with grain size. No strong regularity is observed in the variation of the density of LAGBs with global strain. In small-sized and large-sized grains, the LAGB content first increased and then decreased, whereas, in medium-sized grains, it first decreased and then increased. This is likely because when the tensile deformation is approximately 6%, the medium-sized grains mainly coordinate the continuity of plastic deformation by deflection, whereas the small-sized and large-sized grains mainly coordinate the continuity of deformation by slipping.

## 4. Conclusions

(1) The deformation-induced concave and convex features simultaneously and continuously appear in the surface morphology and have shown a significant inhomogeneity of deformation. The widths of the concave features gradually expand, whereas the widths of the convex features reduce in size. This difference in trend is one of the causes of inhomogeneous deformation.

(2) The size variations of the concave and convex features have been restricted by the deformation coordination of grains and caused a surface roughness (*R_a_*) evolution that first increased and then remained unchanged with increasing stretching strain.

(3) During the stretching process, the large-sized grains in the 270–444 μm range exhibit severe strain localization and the most unfavorable deformation coordination ability, and the small-sized grains in the 37~102 μm range show the most favorable coordination of deformation. The deformation mode of medium-sized grains in the 114–270 μm range tends to grain deflection, and that of others tends to slip.

Future work should elucidate the mechanism of surface roughening under biaxial stress from a deformation perspective by investigating the effect of strain paths on surface roughening behavior.

## Data Availability

The data presented in this study are available upon request from the corresponding author.
